# Life-Threatening Pharyngolaryngeal Hematoma in a Patient With Hemophilia A

**DOI:** 10.7759/cureus.83147

**Published:** 2025-04-28

**Authors:** Kenichi Yanagihara, Yasutaka Mochizuki, Atsushi Katsube, Takuya Kamio, Masaharu Akiyama

**Affiliations:** 1 Department of Otorhinolaryngology, Jikei University School of Medicine, Tokyo, JPN; 2 Division of Clinical Oncology and Hematology, Department of Internal Medicine, Jikei University School of Medicine, Tokyo, JPN; 3 Department of Pediatrics, Jikei University School of Medicine, Tokyo, JPN

**Keywords:** airway obstruction, hematoma, hemophilia, tracheotomy, laryngoscopy

## Abstract

Although airway hematomas are a rare complication of hemophilia, emergency treatment is required in these cases since airway obstruction can cause asphyxia. While the main treatment for hematomas in the airway entails replacing the coagulation factor products, in cases with a high risk of asphyxia, deciding whether or not to perform a tracheotomy can be difficult. We report the case of a patient with severe hemophilia A with rapidly worsening dyspnea due to hematomas caused by an acute upper respiratory tract infection and covering the airway at the upper part of the epiglottis and the tongue base. Laryngoscopy revealed a hematoma of the tongue base, but the hypopharynx and larynx were not visible owing to the hematoma. A CT scan also showed narrowing of the airway due to the hematoma. Given the high risk of obstruction, a tracheotomy was performed under general anesthesia. Efraloctocog alfa, a recombinant coagulation factor VIII (FVIII) product with an extended half-life, was administered to maintain trough levels of coagulation FVIII at 80% or above for eight days after tracheotomy. A repeat CT scan performed four days after the tracheotomy confirmed that the hematoma had improved. The patient was discharged 18 days after the tracheotomy with the tracheostoma closed. If patients with hemophilia complain of throat discomfort or dysphagia, the airway should be assessed via laryngoscopy. Emergency tracheotomy and treatment with a recombinant coagulation factor product by a medical team comprising emergency physicians, hematologists, and otolaryngologists is required for hemophilia patients with a high risk of airway obstruction.

## Introduction

Hemophilia A is a congenital coagulation disorder caused by X-linked recessive inheritance, where the coagulation reaction is delayed due to decreased activity of coagulation factor VIII (FVIII), resulting in symptoms of bleeding [1]. Although the risk of bleeding has decreased with the use of regular replacement therapy with recombinant FVIII, bleeding can still be severe. A type of severe bleeding is intracranial hemorrhage; although a hematoma from the mouth to the pharynx is rare, it can cause airway obstruction [2]. In cases with airway hematoma, physicians must quickly decide whether to perform coagulation factor replacement therapy or to secure the airway via endotracheal intubation or tracheotomy, based on the patient's breathing status, immediately and possibly later. We discuss the case of a patient with severe hemophilia A in whom an emergency tracheotomy was required because of rapidly worsening dyspnea due to an upper respiratory tract infection and a hematoma of the airway from the upper epiglottis to the tongue base.

## Case presentation

**Figure 1:**
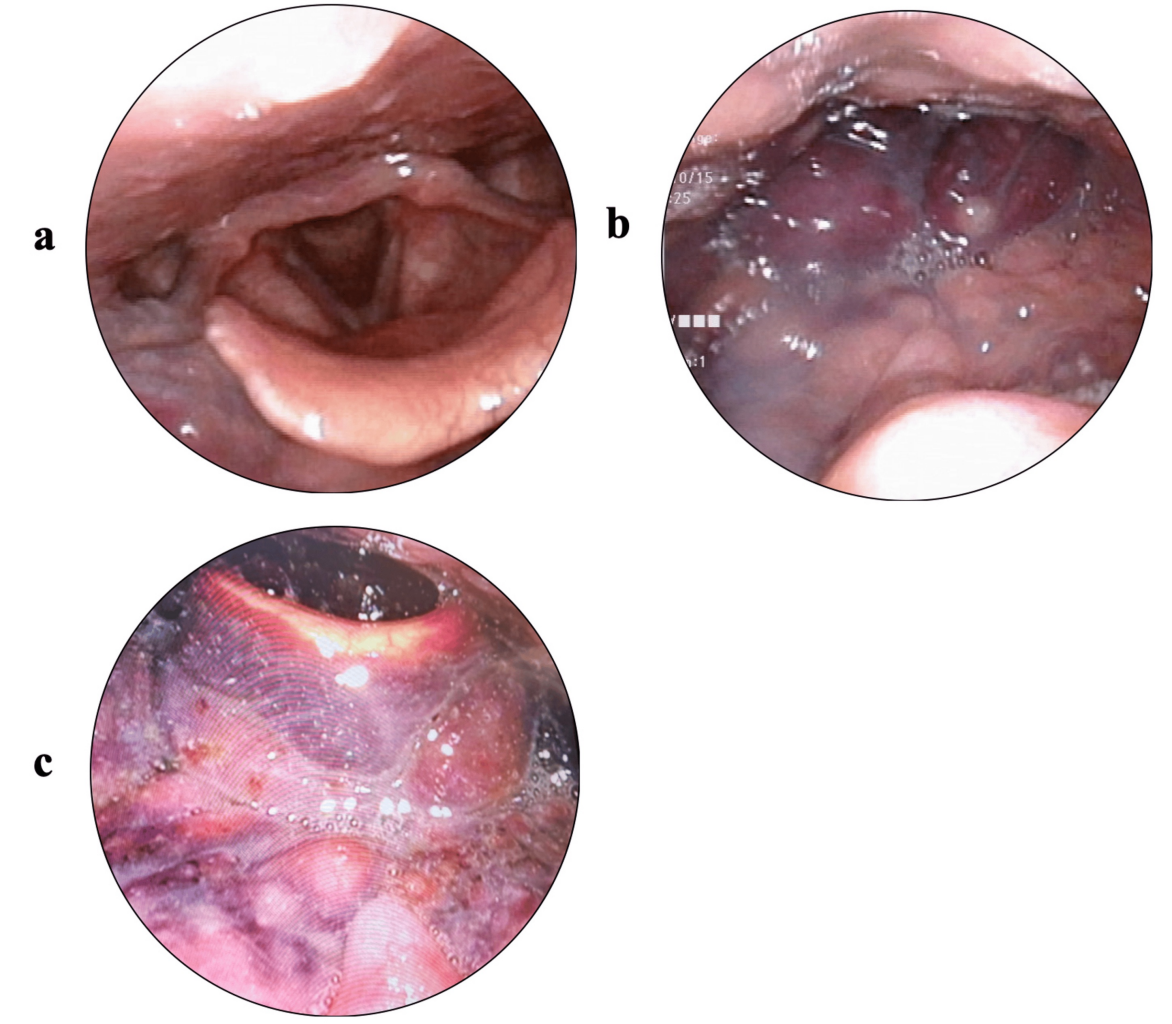
Laryngoscopic images (a) Four days before admission; (b) on the day of admission; and (c) the day after the operation

A 27-year-old male presented with a complaint of rapidly worsening dyspnea. The symptoms had begun with the patient having a fever for three days. Six days later, he had developed a cough, sore throat, and dyspnea and visited a nearby ENT clinic. Although a laryngoscopy had shown no abnormalities (Figure 1a), medication including clarithromycin, L-carbocisteine, and acetaminophen had been prescribed for an upper respiratory tract infection. Two days later, the patient had continued to have dyspnea as well as a fever of 38 °C. On the evening of the next day, he had again visited the same ENT clinic owing to his dyspnea worsening. Hematomas had been found in the upper part of the epiglottis and at the tongue base (Figure 1b); hence, he had been transferred to the emergency medicine department of our hospital at 11:00 p.m. and admitted due to concerns of airway obstruction.

The patient’s medical history included severe hemophilia A, which was diagnosed immediately after his birth. Owing to an intracranial hemorrhage at the age of five months, he had undergone two operations to remove hematomas; however, he had continued to experience right-hand motor impairment. Moreover, he had suffered from an intra-abdominal hemorrhage at the age of 21, and a mediastinal hemorrhage and right thigh muscle hemorrhage when he was 24 years old. Although the replacement therapy for FVIII required that the patient self-administer 2,000 U (35 U/kg) of the efraloctocog alfa, a recombinant coagulation FVIII product with an extended half-life, twice per week, he often failed to secure a blood vessel and had recently administered it only once per week.

**Figure 2:**
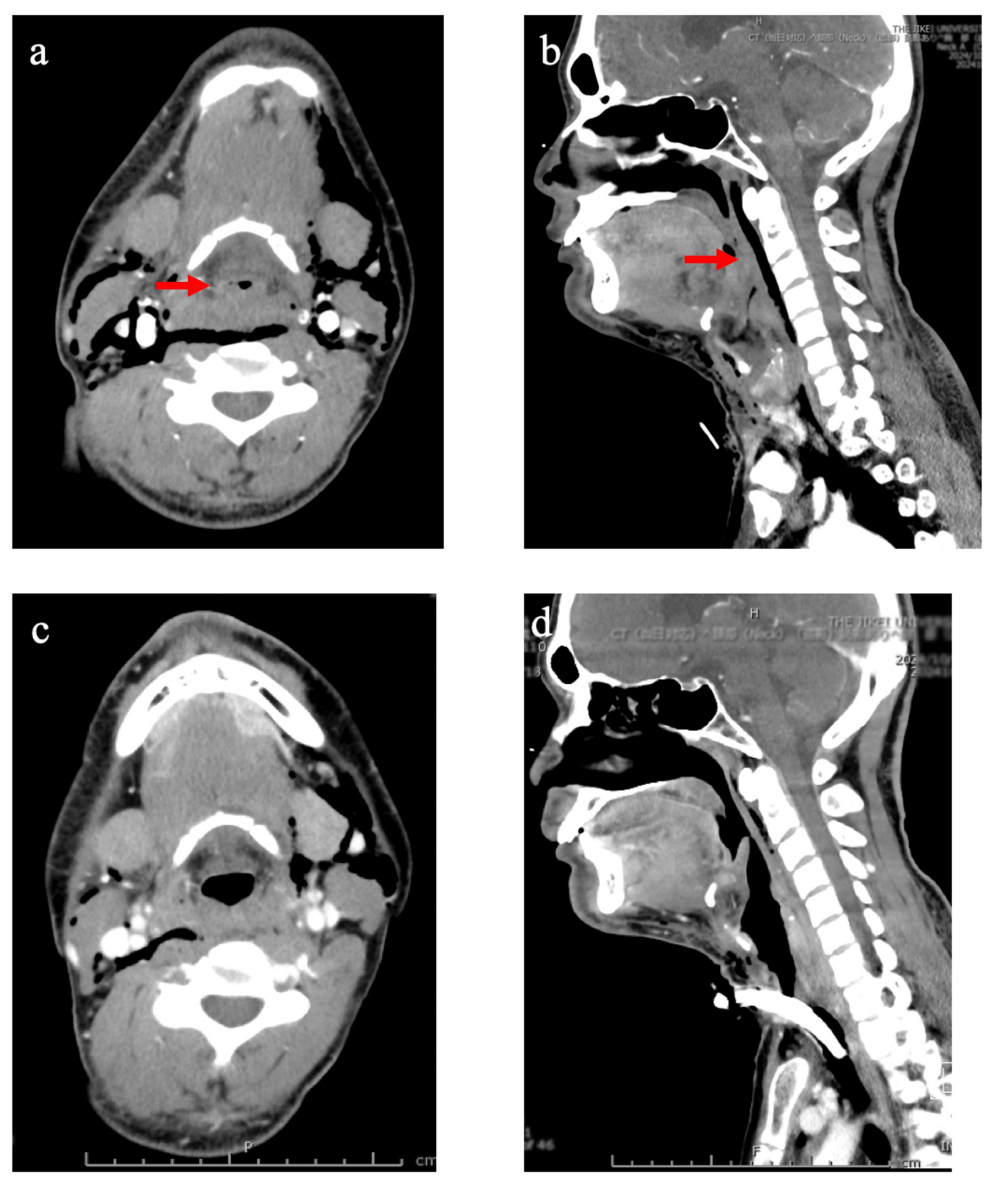
Enhanced CT images of the neck (a) Horizontal image and (b) sagittal CT image performed in the emergency room on the day of admission, and (c) horizontal CT image and (d) sagittal CT image four days after the operation. The red arrow indicates narrowing of the airway due to the hematoma. Edematous swelling suggestive of hematoma was observed in the epiglottis, bilateral aryepiglottic folds, and arytenoid regions; however, no obvious extravasation was identified. The airway was diffusely narrowed at the oropharyngeal, hypopharyngeal, and laryngeal levels (a and b). However, edematous swelling was present, but showed a trend toward improvement (c and d) CT: computed tomography

Physical examination revealed clear consciousness, no cyanosis, and his vital signs were as follows: body temperature of 37.2 °C, a respiratory rate of 18/minute, a heart rate of 96/minute, a blood pressure of 124/72 mmHg, and a saturation of percutaneous oxygen of 98%. The patient had difficulty speaking and felt “something was stuck” in his throat. Since his dyspnea worsened when he was in the supine position, he lay on his side. Laryngoscopy revealed a marked hematoma of the tongue base, but the hypopharynx and larynx could not be observed. Furthermore, an enhanced CT scan showed that the airway was narrowed owing to the hematoma (Figures 2a, 2b). Laboratory data on admission showed an elevated white blood cell count at 14.1 x 10^4^/μL and C-reactive protein at 4.68 mg/dL, but neither anemia (hemoglobin concentration: 15.9 g/dL) nor thrombocytopenia (platelet cell count: 45.0 x 10^4^/μL). Blood gas analysis of venous blood showed the following values: pH: 7.424; PCO_2_: 44.8 mmHg; PO_2_: 22.5 mmHg; cHCO_3_-: 29.2 mmol/L; base excess: 4 mmol/L; and O_2_ saturation: 37.6%. The activated partial thromboplastin time was elevated at 90.4 seconds (reference value: 24-36 seconds), but FVIII activity could not be measured at night. FVIII inhibitor was not found with a test performed during hospitalization.

The otolaryngologist consulted with a hematologist, and the patient was scheduled to undergo an emergency cricothyrotomy because the hematoma was suspected to be rapidly enlarging and blocking the airway. The decision was based on the fact that when the LEMON (Look-Evaluate-Mallampati-Obstruction-Neck mobility) assessment method [3], a multivariate scoring system for assessing the difficulty of endotracheal intubation, was applied to the patient, three factors - Mallampati class III, obstruction, and neck mobility - were identified as issues, and difficulties in intubation were predicted. In the emergency room, a cricothyrotomy was performed while the bleeding was carefully cauterized with a bipolar coagulation hemostatic forceps (hereinafter referred to as “bipolar”) with the patient in a sitting position without his neck being extended. A cuffless endotracheal tube with an internal diameter of 5.0 mm was inserted to secure the airway. However, because this tube could not be left in place for more than 24 hours, the decision was made to perform a tracheotomy.

After the patient was brought to an operating room, the existing tube was replaced with a 6.0-mm cuffed endotracheal tube via a tube exchanger. Under general anesthesia, a tracheotomy was performed. We chose a midtracheal incision and used a large amount of absorbable thread to suture the trachea to the thyroid gland. We were also careful to stop any bleeding that might occur after the operation. Owing to delays in preparation, the patient was treated with 3,000 U of efraloctocog alfa immediately after the operation. Given concerns about infection of the hematoma, we administered ampicillin-sulbactam until the sixth day after the operation. To replenish FVIII activity to a trough level of at least 80%, 3,000 U of efraloctocog alfa was administered twice daily for three days after the operation (Table 1).

**Table 1:** Clinical course of the patient *3000U once per day; **3000U twice per day APTT: activated partial thromboplastin time; CT: computed tomography; ENT: Ear, Nose, and Throat; ER: emergency room; FVIII: factor VIII; r: recombinant

Hospitalization days	Outpatient/inpatient	Fever	Cough	Sore throat	Dyspnea	Imaging studies	Treatments	APTT (second)/FVIII activity (%)
-12		+						
-11		+						
-10		+						
-9								
-8								
-7								
-6								
-5								
-4	ENT clinic		+	+	+	Laryngoscopy (Figure 1a)	Medication	
-3			+	+	+		Medication	
-2		+	+	+	+		Medication	
-1		+	+	+	+		Medication	
1	ENT clinic/ER in our hospital	+	+	+	+	Laryngoscopy (Figure 1b)/CT (Figures 2a, 2b)	Medication	90.4/-
2			+				Tracheotomy/rFVIII replacement*/antibiotics	49.1/-
3			+			Laryngoscopy (Figure 1c)	rFVIII replacement**/antibiotics	41.5/128
4			+				rFVIII replacement**/antibiotics	41.2/149
5			+			CT (Figures 2c, 2d)	rFVIII replacement**/antibiotics	40.7/194
6			+				rFVIII replacement*/antibiotics	
7			+				rFVIII replacement*/antibiotics	41.1/105
8							rFVIII replacement*	44.2/106
9								
10							rFVIII replacement*	49.8/53
11								
12							rFVIII replacement*	51.3/30
13								
14								
15							rFVIII replacement*	58.0/15
16							rFVIII replacement*	
17							Closed trachemostoma	
18							rFVIII replacement*	58.0/14
19								
20	Discharge							

A laryngoscopy the day after the operation showed that the hematoma had become slightly smaller (Figure 1c). Moreover, a CT scan performed three days after the operation showed that the hematoma had become smaller owing to the FVIII replacement therapy (Figures 2c, 2d). Thereafter, over two weeks, the same dose of efraloctocog alfa was changed from once daily to once every other day. The tracheostoma was closed 15 days after the operation. Finally, the patient was discharged 18 days after the operation. There were no abnormalities in vital signs or oxygenation monitoring during the postoperative course. A follow-up examination at the outpatient clinic two weeks after discharge confirmed that the tracheostoma had closed and there were no abnormalities in swallowing or speech.

## Discussion

We described the case of a patient with severe hemophilia A who underwent an emergency tracheotomy due to a hematoma obstructing the airway. Most reports of airway hematomas in patients with hemophilia are related to coagulation factor inhibitors [4]. The airway hematoma in our patient was believed to be due to the following two factors: 1) a difficulty in securing blood vessels for replacement therapy with efraloctocog alfa, resulting in a change in administration from twice a week to once a week and a decrease in FVIII activity, and 2) mucosal damage due to upper respiratory tract infection. Retropharyngeal hematoma due to a medication with an inhibitory or anticoagulant effect has been reported [5], but our patient was not receiving such a drug.

As per a meta-analysis published in 2015, there is no established consensus regarding airway management for upper airway hematoma: management options include conservative treatment, surgical evacuation of the hematoma, oral intubation using a laryngoscope, nasal intubation, fiberoptic-guided tracheal intubation, cricothyrotomy, and tracheotomy [6]. However, the success rates of these individual approaches in managing upper airway hematoma have not been clearly reported. The World Federation of Hemophilia Guidelines for the Management of Hemophilia [7] state that coagulation factor replacement should be performed if hematomas obstruct the airway. Such hematomas have reportedly been improved following coagulation factor replacement [8,9], but tracheostomy has also been performed in these patients [4,10,11].

In our patient, we promptly decided to perform a tracheotomy owing to an airway hematoma with a high risk of asphyxia. Our conclusion that the airway hematoma posed such a risk was based on speech being difficult, neck extension in the sitting position or in the supine position being distressing and difficult, and a significant hematoma of the tongue base observed via fiberoptic bronchoscopy, which made the hypopharynx and larynx impossible to confirm. Although we also considered tracheal intubation if the airway narrowing continued or worsened owing to the tracheal intubation procedure, securing a view of the incision site would be difficult owing to the bleeding tendency associated with poorly controlled hemophilia, and saving the patient’s life might also have been difficult. We decided to first perform a cricothyrotomy and subsequently a tracheotomy.

The LEMON assessment method [3] was also useful for evaluating the necessity of tracheostomy in our patient. When the LEMON method was applied to the present case, three factors were identified and predicted difficulties in intubation. According to a meta-analysis of the LEMON assessment method [12], when a cutoff value of ≥2 criteria was used, the sensitivity was 0.58, specificity was 0.85, and the area under the curve was 0.8698, suggesting that the LEMON method is useful as a multivariate scoring system for assessing the difficulty of endotracheal intubation. Furthermore, in studies comparing the intubation difficulty scale criteria and the LEMON method, neck mobility was particularly identified as an independent predictor of intubation difficulty [13].

The patient's vital signs and oxygenation monitoring upon arrival at the emergency room were within normal ranges. Yamamoto et al. have described a 16-year-old male with hemophilia A in whom a retropharyngeal hemorrhage developed following an upper airway infection: the patient’s vital signs and oxygenation were normal until just before respiratory status suddenly deteriorated [11]. Endotracheal intubation was attempted but was unsuccessful. Cardiopulmonary resuscitation was performed while an emergency percutaneous cricothyrotomy was conducted to secure the airway, followed by endotracheal intubation via a nasal bronchoscope. These emergency procedures took 40 minutes. Therefore, relying solely on vital signs and oxygenation for assessing respiratory status in patients who have hemophilia is considered unsafe.

As a severe complication of hemophilia, airway hematoma is less recognized than intracranial hemorrhage. In our patient, repeated laryngoscopy during an upper airway infection examination at an ENT clinic enabled prompt diagnosis of an airway hematoma. This case highlights the importance of collaboration with hematologists and otolaryngologists when patients with hemophilia present with pharyngeal discomfort or dyspnea; however, establishing such a collaborative framework remains a challenge. Additionally, to evaluate the usefulness of LEMON in assessing respiratory status in patients with hemophilia who have airway hematoma, further reports of similar cases are necessary.

## Conclusions

Airway obstruction due to hematomas is a serious complication in patients with severe hemophilia and can be life-threatening. Therefore, emergency physicians, hematologists, and otolaryngologists must work together to assess the patient’s breathing condition and take appropriate and prompt action based on this assessment. Furthermore, to prevent airway obstruction due to hematomas in such patients, it is important to adequately manage regular replacement of coagulation factor products and assess the airway for respiratory tract infections by laryngoscopy. The findings of this article are limited as they are derived from a single case. However, we believe that the experience of physicians in treating patients with hemophilia and airway hematoma will contribute to a better understanding of these findings.
